# Predicting formation of adhesions after gynaecological surgery: development of a risk score

**DOI:** 10.1007/s00404-015-3804-0

**Published:** 2015-07-30

**Authors:** Per Lundorff, Hans Brölmann, Philippe Robert Koninckx, Michal Mara, Arnaud Wattiez, Markus Wallwiener, Geoffrey Trew, Alison M. Crowe, Rudy Leon De Wilde, For the Anti-Adhesions in Gynaecology Expert Panel (‘ANGEL’)

**Affiliations:** Department of Gynaecology, Private Hospital Molholm, Vejle, Denmark; Department of Obstetrics and Gynaecology, VU University, Amsterdam, The Netherlands; Department of Obstetrics and Gynaecology, University Hospital Gasthuisberg, Katholieke Universiteit, Leuven, Belgium; Department of Obstetrics and Gynaecology, Charles University, Prague, Czech Republic; Department of Obstetrics and Gynecology, Hôpital de Hautepierre, Strasbourg, France; Department of Obstetrics and Gynaecology, University of Heidelberg, Heidelberg, Germany; Department of Reproductive Medicine and Surgery, Hammersmith Hospital, London, UK; Corvus Communications Limited, Buxted, East Sussex UK; Department of Gynecology, Obstetrics and Gynecological Oncology, University Hospital for Gynaecology, Pius-Hospital, University Oldenburg, Oldenburg, Germany

**Keywords:** Adhesions, Risk, Surgery, Complications, Laparoscopy

## Abstract

**Purpose:**

Risk factors for post-surgical adhesions following gynaecological surgery have been identified, but their relative importance has not been precisely determined. No practical tool exists to help gynaecological surgeons evaluate the risk of adhesions in their patients. The purpose of the study was to develop an Adhesion Risk Score to provide a simple tool that will enable gynaecological surgeons to routinely quantify the risk of post-surgical adhesions in individual patients.

**Methods:**

A group of European gynaecological surgeons searched the literature to identify the risk factors and the surgical operations reported as carrying a risk of post-surgical adhesions. Through consensus process of meetings and communication, a four-point scale was then used by each surgeon to attribute a specific weight to each item and collective agreement reached on identified risk factors and their relative importance to allow construct of a useable risk score.

**Results:**

Ten preoperative and 10 intraoperative risk factors were identified and weighed, leading to the creation of two sub-scores to identify women at risk prior to and during surgery. The Preoperative Risk Score can range from 0 to 36, and the Intraoperative Risk Score from 3 to 31. Several thresholds between these limits may be used to identify women with low, medium, and high risk of post-surgical adhesions.

**Conclusions:**

Gynaecological surgeons are encouraged to use this Adhesion Risk Score to identify the risk of adhesions in their patients. This will allow better informed use of available resources to target preventive measures in women at high risk of post-surgical adhesions.

## Introduction

Postoperative adhesions are a frequent complication of abdominopelvic surgery [1, 2]. Adhesions complicate future surgery, extending length of surgery and posing serious risks to the patient, particularly bowel injury; and they cause adhesion-related disease [3, 4]. They are important causes of chronic abdominal pain and dyspareunia [1], are the leading cause of secondary infertility in women, accounting for 20–40 % of all cases of female infertility [5, 6], and a lifetime risk of small-bowel obstruction [1]. The impact of adhesions on the quality of life of patients is considerable, but often overlooked [4].

Despite advances in surgical techniques, the healthcare burden of adhesion-related complications has not changed in recent years [1, 7–10]. While numbers of adhesions forming may be reduced following laparoscopic surgery [11], adhesion-related complications still remain, and for most therapeutic gynaecological laparoscopic procedures, the comparative risk of adhesion-related complications is similar to gynaecological laparotomy [7, 12]. Population-based epidemiological research has demonstrated that some types of gynaecological surgery put patients at higher risk of adhesion-related complications [12–14].

The mainstay treatment for adhesions is adhesiolysis. However, reformation of adhesions occurs in most patients (mean 85 %) regardless of the method of adhesiolysis used or the type of adhesion being lysed [7].

Intraoperative use of adhesion–reduction agents is based on the rationale that contact between two areas of injury is necessary to form an adhesion. These agents act as barriers between injured areas, significantly reducing the development of adhesions [15–20]. However, due to the associated added costs, which are high for some agents, it may be more economical to target women at high risk of adhesions and associated complications [15, 24, 28].

Apart from population-based epidemiological research [12–14, 21–23], the published literature gives a limited guide to specifically identifying who is at most risk, and most studies have looked at the global rate of adhesion formation after abdominal and not specifically, gynaecological surgery. The accepted rate of post-gynaecological surgery adhesions is imprecise, varying from 55 to over 90 % [1].

A consistent method to identify women at high risk of adhesions following gynaecological surgery is currently lacking. This prompted us to progress this expert consensus project to collectively review the literature with our own experience as gynaecological surgeons, to develop a consensus-based Adhesion Risk Score.

## Methods

Members of the consensus expert panel designed and agreed on the process described hereafter.

As the first step, systematic reviews, randomised control trials/controlled clinical trials, cohort studies and meta-analyses published in English specifically addressing postoperative adhesions, adhesion prevention, and adhesion–reduction agents were searched via Medline using key words—post-surgical adhesions, abdominal adhesions, peritoneal adhesions and gynaecological surgery adhesions. No time limit on publication was employed.

During a first meeting, the expert panel collectively identified and agreed the risk factors for post-surgical adhesions reported in the literature, and divided them into preoperative, intraoperative, and postoperative risk factors.

A second meeting determined the precise wording of risk factors and, where applicable, the values and thresholds of numerical variables. The meeting decisions were then circulated within the panel and each member was asked to rate each risk factor using a scale from 1 (low risk of adhesions) to 4 (very high risk of adhesions).

During the third and last round of the consensus process, discrepancies regarding the relative weight of each factor were resolved through face-to-face meetings or phone/e-mail discussions. Following field testing of the scoring in routine practice in ~ 40 patients at 4 centres, thresholds for low, medium, and high risk of post-surgical adhesions were determined at a final meeting to ratify the project.

## Results

### Literature data selected

From review of the emerging literature, it was clear that among the published epidemiological and clinical studies, many dealt with adhesions following abdominal surgery in general or non-gynaecological interventions such as colorectal surgery; others were dedicated to complications of adhesions such as small bowel obstructions. Such studies were not considered relevant for our purpose. Those specifically addressing adhesions following gynaecological surgery were comparatively few.

During the first meeting, we agreed that the most valuable sources of information on risk factors were several consensus position papers [15, 24–27], the wide-scale epidemiological study SCAR-2 [12], and a systematic review published by the Society of Obstetricians and Gynaecologists of Canada [28].

The SCAR-2 study was a retrospective assessment of adhesion-related readmissions in 24,046 patients undergoing gynaecological laparotomic or laparoscopic surgery. This study demonstrated that the risk of adhesion formation, and subsequent complications, varied depending on the anatomical site of the intervention, at least if the laparotomic method had been chosen.

We collectively agreed the risk factors for post-surgical adhesions reported in the literature, taking into account known risks due to patient history, surgical technique, side effects of the operation and the anatomical sites of gynaecological surgery—and sub-divided them into preoperative, intraoperative, and postoperative risk factors.

Analysis of the selected publications revealed general consensus on several other risk factors, including the total number of abdominal and pelvic surgeries a patient underwent, bleeding, tissue trauma, and the use of foreign materials. However, there was no indication in the literature of the risk level inherent to these risk factors.

### Adhesion Risk Score

We reviewed the literature data with our own clinical experiences, following the successive steps of the process described in the “Methods” section.

A second meeting determined the precise wording of risk factors and, given the paucity of published evidence, we assigned risk values and thresholds of numerical variables. We agreed on a list of risk factors which were considered simple to assess in routine clinical practice and whose relative importance we rated from one to four.

Seven preoperative, ten intraoperative, and three postoperative risk factors were retained. After discussion, we decided that for clinical utility, the Adhesion Risk Score should be simply divided into two sub-scores, namely the Preoperative Adhesion Risk Score; incorporating the identified Postoperative Risk Factors and ‘risk factors associated with previous operations’; and the Perioperative Adhesion Risk Score. These constitute the total Adhesion Risk Score (ARS) shown in Table 1.

**Table 1:** Adhesion Risk Scores proposed to estimate risk of post-surgical adhesions in women undergoing gynaecological surgery

Risk factors			Assigned Score
a) Preoperative Adhesion Risk Score			
Preoperative risk factors	Previous abdominal/pelvic surgery	1	3
		>1	4
	History of post-surgical adhesions		4
	Concomitant abdominal or gynaecological inflammation and/or infection		4
	Endometriosis	Minimal	1
		Mild	2
		Moderate	3
		Severe	4
	Cancer	Gynaecologic cancer	2
		Peritoneal carcinomatosis	2
		Local non-gynaecological cancer	3
		Metastatic cancer of extrapelvic origin	2
	Radiation therapy in intra-abdominal cancer	Local	4
		Distant sites	1
	Keloid scarring		3
Risk factors associated with previous operations	Intraperitoneal bleeding	Unexpected 2 g % drop of Hb	2
	Postoperative complications e.g. fistulas, abscesses		4
	Postoperative Infection (≥38 °C for ≥2 days)		4
		Total	
b) Perioperative Risk Score			
Perioperative risk factors	Quality of existing adhesions	None	0
		Filmy	2
		Vascular	3
		Dense	4
	Severity of existing adhesions	No adhesions	0
		Single adhesion	1
		2 or 3 adhesions	2
		>3 adhesions	3
		Adhesion(s) with bowel involvement	4
	Bleeding	>500 ml	4
	Procedure duration	<90 min	2
		9 min to 2 hours	3
		>2 hours	4
	Procedure complexity or extent of surgery e.g. enterotomy, oncological surgery		3
	Multiple quadrants e.g. adhesiolysis, ovarian carcinoma surgery		
	Excessive coagulation >2 cm^2^		2
	Type and site of surgery	Laparoscopy/Fallopian tube	1
		Open/Uterus	3
		Open/Fallopian tube	2
		Laparoscopy/Adhesiolysis, uterus	3
		Laparoscopy/All other procedures	2
		Open/Ovary	4
	Intra-abdominal placement of foreign bodies e.g. meshes		3
	Use of electrical scalpel		2
	Peritoneal closing		1
		Total	

Two widely accepted classifications published in the literature were used to facilitate the rating of two risk factors. For the Preoperative Adhesion Risk Score, we used the Revised American Society for Reproductive Medicine classification of endometriosis to determine whether the severity of endometriosis, if present, falls into the minimal (rated one), mild (rated two), moderate (rated three) or severe category (rated four) [29]. For the Perioperative Adhesion Risk Score, the four possible rates specified to determine the severity of pre-existing adhesions—single adhesion, two or three adhesions, >3 adhesions, and adhesion(s) with bowel involvement—correspond to the categories that were originally defined by Knightly et al. [30], and which have been adopted as standard over time.

Meeting decisions were circulated within the panel and each member rated each risk factor using a scale from one (low risk of adhesions) to four (very high risk of adhesions).

### Thresholds for low, medium, and high risk of post-surgical adhesions

Tables 2 and 3 illustrate that the Preoperative Adhesion Risk Score can range from zero to 36 and the Intraoperative Adhesion Risk Score from three to 31 in individual women undergoing gynaecological surgery. We used the tertiles of these ranges to provide an initial approach using the thresholds of low, medium, and high risk of adhesion formation. After evaluating the predictive value of those thresholds in our field testing in a limited series of women, we propose the adhesion risk levels illustrated in Table 4.

**Table 2:** Minimum and maximum possible Preoperative Adhesion Risk Score achievable in women undergoing gynaecological surgery

Risk Factors			Assigned Score
a) Minimum Preoperative Risk Score			
Preoperative risk factors	Previous abdominal/pelvic surgery	0	0
	History of post-surgical adhesions	Absent	0
	Concomitant abdominal or gynaecological inflammation and/or infection	Absent	0
	Endometriosis	Absent	0
	Cancer	Absent	0
	Radiation therapy in intra-abdominal cancer	Absent	0
	Keloid Scarring	Absent	0
Risk factors associated with previous operations	Intraperitoneal bleeding	Absent	0
	Postoperative complications e.g. fistulas, abscesses	Absent	0
	Postoperative Infection (≥38 °C for ≥2 days)	Absent	0
		Total	0
b) Maximum Preoperative Risk Score			
Preoperative risk factors	Previous abdominal/pelvic surgery	>1	4
	History of post-surgical adhesions	Yes	4
	Concomitant abdominal or gynaecological inflammation and/or infection	Yes	4
	Endometriosis	Severe	4
	Cancer	Local non-gynaecological cancer	3
	Radiation therapy in intra-abdominal cancer	Local	4
	Keloid Scars	Yes	3
Risk factors associated with previous operations	Intraperitoneal bleeding	Unexpected 2 g % drop of Hb	2
	Postoperative complications *e.g. fistulas, abscesses*	Yes	4
	Postoperative Infection (≥38 °C for ≥2 days)	Yes	4
		Total	36

**Table 3:** Minimum and maximum possible Perioperative Adhesion Risk Score achievable in women undergoing gynaecological surgery

Risk Factors			Assigned Score
a) Minimum Perioperative Risk Score			
Intraoperative risk factors	Quality of existing adhesions	None	0
	Severity of adhesions	None	0
	Procedure duration	<90 min	2
	Type and site of surgery	Laparoscopy/Fallopian tube	1
		Total	3
b) Maximum Perioperative Risk Score			
Intraoperative risk factors	Quality of existing adhesions	Dense	4
	Severity of adhesions	Adhesion(s) with bowel involvement	4
	Bleeding	>500 ml	4
	Procedure duration	>2 hours	4
	Procedure complexity or extent of surgery e.g. enterotomy, oncological surgery Multiple quadrants e.g. adhesiolysis, ovarian carcinoma surgery		3
	Excessive coagulation >2 cm^2^		2
	Type and site of surgery	Open/Ovary	4
	Intra-abdominal placement of foreign bodies e.g. meshes		3
	Use of electrical scalpel		2
	Peritoneal closing		1
		Total	31

**Table 4:** Ranges and thresholds of low, medium, and high risk of formation of post-surgical adhesions

Preoperative Risk Score		Perioperative Risk Score	
Low risk	0–12	Low risk	3–17
Medium risk	13–24	Medium risk	18–28
High risk	25–36	High risk	29–31

## Discussion

The Adhesion Risk Score presented here was developed from comprehensive searching of the literature and review of pertinent publications, and expert consensus process. It is the first practical tool proposed for gynaecological surgeons to use in their routine surgical practice to evaluate the risk of post-surgical adhesions in individual patients.

The two sub-scores (Preoperative and Perioperative) have a similar objective—to help surgeons identify women at particular risk of post-surgical adhesions in a consistent fashion, and from this to make better informed decisions on targeting use of adhesion–reduction agents where resources limit their ability to use them widely.

The Preoperative Adhesion Risk Score may be calculated to evaluate the individual adhesion risk level specific to each woman prior to any gynaecological operation. This should thus help the surgeon to adapt the surgical technique as necessary, and decide whether the woman should receive an adhesion–reduction agent, and which one—considering the type of pathology and surgical procedure to be undertaken. No less importantly, it also reminds the surgeon of the necessity to ensure that patients are informed of the potential risks of adhesions before their surgery, thus not only fulfilling their duty of care, but also avoiding potential for medicolegal litigation [15, 26]. The Preoperative Adhesion Risk Score can be simply adopted into use as part of the routine preoperative assessment and informed consent process.

As well as potentially identifying further increased risk, the Perioperative Adhesion Risk Score may also help identify those women who may appear to have a low Preoperative Adhesion Risk Score, but who are nevertheless at considerable risk of adhesion formation because of specific risk factors directly linked to the operation process and/or to the characteristics of pre-existing adhesions not known on preoperative assessment. In those women who are then identified as at high risk, the use of an anti-adhesion agent would not only be of likely clinical benefit but also more easily economically justifiable where resources are limited.

Although calculating the Perioperative Adhesion Risk Score during surgery may seem impractical, this can be simply addressed by having the Perioperative Adhesion Risk Score available as a poster or on screen in the operating room as an aide memoire. The score can then simply be calculated without impacting on the duration of the surgical process—only 10 numbers ranging from zero to four need adding together.

Prior to the development of this Adhesion Risk Score, we were aware of the heterogeneity of data on risk factors for adhesions reported in the literature. Indeed, wide variations exist in adhesion classification and surgical approaches, making comparison between the published evidence difficult. This hampered a fully objective determination of the risk level associated with each adhesion risk factor and the development of a properly evidence-based risk score. Alongside a comprehensive review of the literature, the expert panel consensus process provided the most appropriate method to develop the Adhesion Risk Score presented here.

Due to both the heterogeneity and paucity of data on the relative importance of risk factors for adhesions following gynaecological surgery, by consensus, we adopted a simplified scoring process with the weight attributed to each risk factor counted from one to four instead of the one to nine range generally adopted [31].

Within this context, proposing an accurately graded evidence-based evaluation of the risk of post-surgical adhesions in individual women would have been far too ambitious. However, we believe that the three broad categories of low, medium, and high risk proposed should help surgeons better identify women who may benefit most from preventive measures to minimise post-surgical adhesions, providing improved justification and targeting of use of adhesion–reduction agents in healthcare systems where resources and funding are limited.

Cost considerations must be taken into account when deciding whether an adhesion–reduction agent should be used. Healthcare providers do not generally refund the cost of adhesion–reduction agents. Gynaecological surgeons participating in two surveys of adhesions awareness conducted in Germany [32] and in several European countries [33] declared that currently, products were too expensive to be used extensively. These economic factors preclude the routine use of adhesion–reduction agents in gynaecological surgery.

As operating surgeons, we have to better target all our existing resources and while fully recognising the seriousness and extent of the problem of adhesions, a key issue we sadly face is justifying the use of an adhesion–reduction agent. We encourage gynaecological surgeons to use the Adhesion Risk Score to evaluate the risk of adhesions in their patients in a consistent fashion and thus assist in making both better informed decisions and justification for use of appropriate preventive measures in high-risk patients, especially in younger women identified as at high risk of adhesions who wish to conceive.

We acknowledge that our Adhesion Risk Score is a first attempt that may need refinement after testing in broader routine surgical practice. While building an evidence-based risk score using an appropriate statistical method [34] is clearly a desirable goal, it will require more data and more stringent evidence which at present is not available. However, in the meantime, we present here a simple method that can be easily adopted into routine practice to evaluate adhesion risk in a systematic fashion, and thus help improve identification and clinical justification for use of limited resources by targeting women most at risk.
